# Over-representation of specific regions of chromosome 22 in cells from human glioma correlate with resistance to 1,3-bis(2-chloroethyl)-1-nitrosourea

**DOI:** 10.1186/1471-2407-6-2

**Published:** 2006-01-04

**Authors:** Nicole C Hank, Joan Rankin Shapiro, Adrienne C Scheck

**Affiliations:** 1 Neuro-Oncology Research, Barrow Neurological Institute^® ^of St. Joseph's Hospital and Medical Center, 350 W. Thomas Rd., Phoenix, AZ 85013 USA; 2 Neurosurgery Research, Barrow Neurological Institute^® ^of St. Joseph's Hospital and Medical Center, 350 W. Thomas Rd., Phoenix, AZ 85013 USA

## Abstract

**Background:**

Glioblastoma multiforme is the most malignant form of brain tumor. Despite treatment including surgical resection, adjuvant chemotherapy, and radiation, these tumors typically recur. The recurrent tumor is often resistant to further therapy with the same agent, suggesting that the surviving cells that repopulate the tumor mass have an intrinsic genetic advantage. We previously demonstrated that cells selected for resistance to 1,3-bis(2-chloroethyl)-1-nitrosourea (BCNU) are near-diploid, with over-representation of part or all of chromosomes 7 and 22. While cells from untreated gliomas often have over-representation of chromosome 7, chromosome 22 is typically under-represented.

**Methods:**

We have analyzed cells from primary and recurrent tumors from the same patient before and after *in vitro *selection for resistance to clinically relevant doses of BCNU. Karyotypic analyses were done to demonstrate the genetic makeup of these cells, and fluorescent *in situ *hybridization analyses have defined the region(s) of chromosome 22 retained in these BCNU-resistant cells.

**Results:**

Karyotypic analyses demonstrated that cells selected for BCNU resistance were near-diploid with over-representation of chromosomes 7 and 22. In cells where whole copies of chromosome 22 were not identified, numerous fragments of this chromosome were retained and inserted into several marker and derivative chromosomes. Fluorescent *in situ *hybridization analyses using whole chromosome paints confirmed this finding. Additional FISH analysis using bacterial artificial chromosome probes spanning the length of chromosome 22 have allowed us to map the over-represented region to 22q12.3–13.32.

**Conclusion:**

Cells selected for BCNU resistance either *in vivo *or *in vitro *retain sequences mapped to chromosome 22. The specific over-representation of sequences mapped to 22q12.3–13.32 suggest the presence of a DNA sequence important to BCNU survival and/or resistance located in this region of chromosome 22.

## Background

Treatment of human malignant gliomas often includes surgical resection followed by chemotherapy and radiation; however, it is common for such tumors to recur despite adjuvant therapy [1]. The recurrent tumor is often resistant to further therapy with the same agent, suggesting that cells which survive treatment and repopulate the tumor mass have an intrinsic genetic advantage. We have previously demonstrated that cells selected for resistance to 1,3-bis(2-chloroethyl)-1-nitrosourea (BCNU) *in vitro *or *in vivo *(recurrent tumor) were near diploid, with over-representation of part or all of chromosomes 7 and 22 [2]. Whereas over-representation of chromosome 7 is common in gliomas, chromosome 22 is not typically over-represented, and in fact it is often under-represented in untreated gliomas [3-7]. However, when a sufficient number of karyotypes are done it is possible to identify cells with over-representation of chromosome 22 in untreated tumors. These cells represent a very minor proportion of the cells in the primary, untreated tumor, but become a major subpopulation after treatment. We were able to demonstrate that this is likely due to selection of these cells through the identification of karyotypic markers in cells from the primary and recurrent tumors [2]. Thus, selection for cells with over-representation of chromosome 22 sequences by BCNU treatment suggests the presence on this chromosome of a gene or genes that confer a selective advantage to these cells.

We originally analyzed the expression of platelet-derived growth factor because the genes encoding the A and B chains of this growth factor are mapped to chromosome regions 7p22 and 22q13.1, respectively. Whereas we demonstrated increased expression of these genes in some BCNU-resistant cells, it was likely that this over-expression provided a growth advantage and was not directly involved in resistance [8]. We also analyzed the expression of glutathione-*S*-transferase theta 1, a gene mapped to 22q11.23. Over-expression of this gene at the RNA level was not found in the majority of our BCNU resistant cells [9].

The specific over-representation of chromosome 22 sequences provides strong evidence that a gene(s) on this chromosome is important for survival after therapy and/or therapy resistance. The availability of samples from tumors that recurred following therapy with BCNU and radiation provided us with a unique opportunity to examine cells that survived therapy *in vivo*. To assist in the identification of the gene(s) involved in the growth of BCNU resistant cells, we established sets of 4 cell lines from each of three patients (Table 1); cells from the primary tumor, cells from the primary tumor selected for resistance to 10 μg/ml of BCNU *in vitro*, cells from recurrent tumor (*in vivo *selection), and cells from recurrent tumor selected for resistance to 10 μg/ml of BCNU. These cells were analyzed for the retention of chromosome 22 by karyotypic analyses and the specific regions that were retained were identified using fluorescent *in situ *hybridization with bacterial artificial chromosome (BAC) probes.

## Methods

### Cell culture and selection for drug resistant cell populations

The availability of samples from tumors that recurred after therapy provided us with a unique opportunity to examine cells that survived therapy *in vivo*. Patients from whom we have cell lines derived from both primary and recurrent tumor samples were used for this study (Table 1). The time between surgeries and overall survival for patients this age was not unusual. All three patients received gross total resections of their primary tumor and all were then treated with BCNU and radiation. All three showed increased enhancement within 3 months of the primary surgery, demonstrating rapid progression of the tumors. Within a year all three required surgery for recurrent tumor (Table 1).

Primary tumors were given a random 2-letter code designation, and the recurrent tumor from the same patient received the same code with the addition of "R" (ME/MER, LX/LXR, DI/DIR). Cell lines were derived as described [10,11] and grown in Waymouth MAB 87/3 medium (MAB) with 20% fetal calf serum (FCS). The passage number of the cells used for all experiments varied (LX/LXR 15–32, DI 5–25, DIR 15–40, ME 18–41; MER 52–120); however, the results of repeats done at different passage numbers were consistent. Cells were selected for resistance to 10 μg/ml BCNU as described [10,12], and mock treatment of the cells was done in parallel. Briefly, cells were washed with MAB without serum 3 times; they were then mock-treated using MAB without serum or drug, or treated with BCNU in MAB without serum for 1 hour at 37°C with 5% CO_2_. Cells were washed and fed with MAB containing 20% serum. The cells were treated with the same concentration of BCNU for three consecutive days, after which the cells were allowed to grow. This step was repeated several times until the resulting cell population was resistant, as evidenced by the absence of cytopathic effect and cell debris in the media which would indicate cell death after treatment when compared to the mock-treated controls. When cells appeared resistant to a particular dose of BCNU, subsequent treatment was done at a higher dose (2.5, 5.0, 7.5, 10.0 μg/ml) until cells were resistant to 10 μg/ml. The time required to select for a resistant cell population varied for the different cell lines. For example, DI cells required approximately 3 months to get a cell line fully resistant to 10 μg/ml BCNU. Tumor cell lines LX and ME required 4–6 months. Selection for resistant cells from recurrent tumors (DIR, LXR, MER) was complete in 2–3 months for all cell lines, reflecting the higher level of intrinsic resistance in cells from the recurrent tumors compared to cells from the primary tumor. Cells were re-treated with 10 μg/ml BCNU every 8–10 passages to maintain the resistant phenotype.

### Karyotype analysis

Karyotype analysis was done on low passage cells as described previously [13]. Briefly, mitotic cells from mock-treated or BCNU-resistant cultures were harvested by banging the sides of the flasks to release dividing cells into the medium. Cells were pelleted, swelled using 0.075 M KCl at 37°C for 17 min and 22 sec, and fixed with methanol/acetic acid (3:1). Cells were then dropped onto slides, rinsed with methanol/acetic acid, dried, and stored until use [14]. The nomenclature follows ISCN recommendations [15,16]. Chromosomal abnormalities were classified as clonal if two or more metaphases had an identical structural abnormality, or five or more metaphases had gained or lost a specific chromosome. Non-clonal cells represent cells in which unique structural arrangements, telomeric fusions, somatic exchanges and/or different states of chromosome contraction occur.

### Probes for FISH analyses

**Figure 1:**
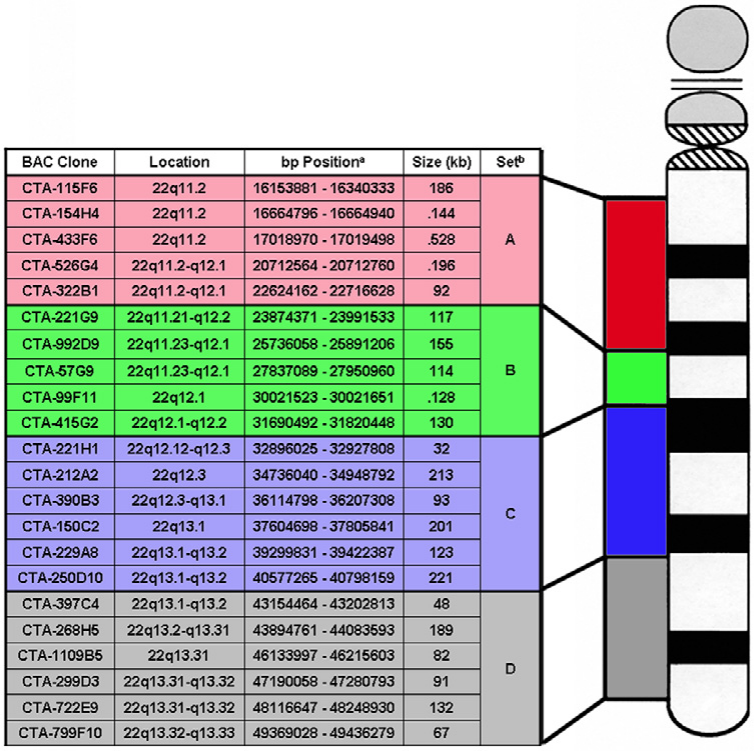
**Chromosome 22 ideogram**. Chromosome 22 ideogram showing the size and location of the individual BACs and the 4 grouped BAC pools used in this study. ^a ^bp position obtained from The Sanger Center; ^b ^set denotes BAC pools.

Whole chromosome paint for chromosome 22 (Vysis, Inc., Downers Grove, IL) was used following conditions specified by the manufacturer. Bacterial artificial chromosomes (BACs) spanning the q arm of chromosome 22 at 2-megabase intervals (2 Mb BAC Set; a kind gift from The Sanger Institute, Cambridge, UK) were used to identify the regions of chromosome 22 retained in BCNU resistant cells (Figure 1). The BACs were grown and purified according to standard protocols [17]. To verify that there was no cross hybridization to other chromosomes, BACs were tested against CGH Metaphase Target Slides which contain metaphases derived from karyotypically normal male cultured lymphocytes (Vysis Inc.). Labeling with SpectrumGreen™ or SpectrumOrange™ was done with a Nick Translation kit (Vysis) under conditions specified by the manufacturer. For identifying the region of chromosome 22 retained in BCNU-resistant cells, BAC DNAs were individually labeled and tested prior to being separated into one of 4 groups (Figure 1). Pooled probes contained 0.1 μg of each BAC DNA, 5 μg salmon sperm DNA, and 6 μg Cot-I DNA. Individual BAC probes contained 0.1 μg of BAC DNA, 5 μg salmon sperm DNA, and 1 μg Cot-I DNA.

To verify the hybridization efficiency of our probes the BACs were separated into 2 groups (Pools A+B and C+D) and tested using CGH Metaphase Target Slides (Vysis). Ninety five percent (102/107 cells counted) of the metaphase spreads were diploid when pool A+B was used as probe and 94% (113/120) were diploid using C+D.

### Cell preparation and FISH analysis

Mitotic cells from mock-treated or BCNU-resistant cultures were harvested by banging the sides of the flasks to release dividing cells into the medium. Cells were pelleted, swelled using 0.075 M KCl at 37°C for 17 min and 22 sec, and fixed with methanol/acetic acid (3:1). Cells were then dropped onto slides, rinsed with methanol/acetic acid, dried, and stored until use [14]. Slides used for FISH were denatured in 70% deionized formamide at 73°C for 5 min, dehydrated, and hybridized with 10 μl hybridization mix consisting of labeled BAC probes in LSI hybridization buffer (Vysis) at 37°C for approximately 20 hours in a Perkin Elmer GeneAmp System 1000 *in situ *hybridization instrument. Slides were then washed in 0.4 × SSC (Vysis) for 2 min at 73°C, rinsed with 2 × SSC at room temperature, and left to air-dry. Preparations were counterstained with propiduim iodide or DAPI, and FISH signals were visualized and captured with a Zeiss Pascal 5 Laser Scanning Confocal Microscope. At least 75 metaphase cells per cell line were examined; chromosome fragments were considered over-represented if three or more copies were seen per cell. Statistical analysis was done using the SAS (Statistical Analysis Software, SAS Institute, Inc, Cary, NC) PROC logistic regression method.

## Results

### Karyotype analysis

**Figure 2:**
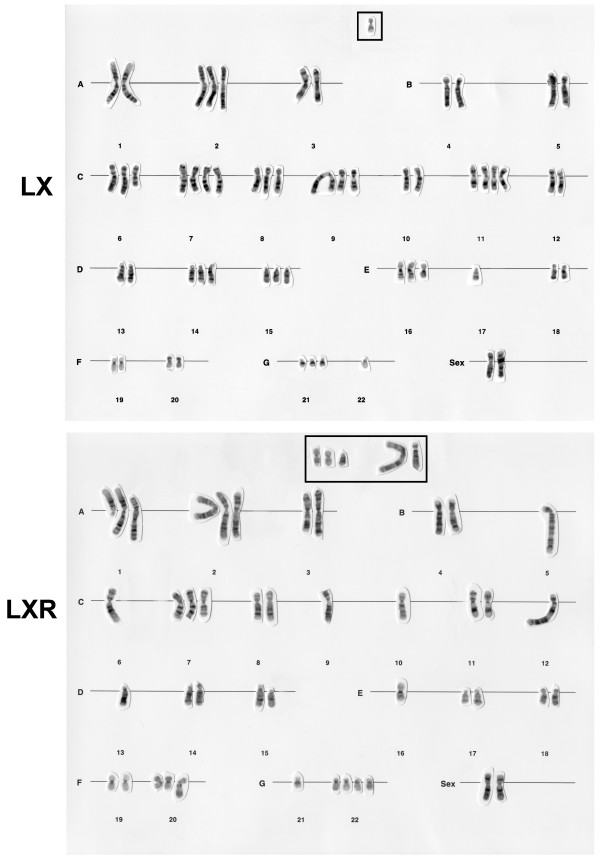
**Karyotypes**. **(A) **Representative karyotype from the primary LX tumor cell passage 2. Karyotypic deviation: 57XX, +2, i(6)(p10), +7, +7, +8, +9, der(9)t(9;12)(p13;q11)ins(9;?)(p13;?), +11, +11, +12, +14, +15, +16, -17, del(17)(p11.2), +21, -22, + mar. **(B) **Representative karyotype from the recurrent LXR tumor cell passage 3. Karyotypic deviation: 49XX, del(1)(q12), +der(2)t(2;9)(p13;q13), der(3)ins(3;?)(p23;?), add(3)(q21), der(4)t(4;8)(q13.2;q11.2), -5, add(5)(q11.1), -6, der(6)t(1;6)(p11;q11), +add(7)(q21.2), -9, add(9)(q32), -10, add(11)(p11.2), -12, add(12)(q24.3), -13, add(15)(q21.2), -16, del(17)(p11.2) ×2, +add(20)(q11.1), -21, +22, +22, +5 mar.

Figure 2A and 2B show representative karyotypes from the primary (A) and recurrent (B) tumors from patient coded LX/LXR and Table 2 gives a summary of the results for all three patients. Karyotype analysis was done on low passage cells to avoid artifacts that could arise from long term *in vitro *cultivation. Primary (passage 0) cells from tumors DI, DIR, ME and LX were prepared and analyzed as described in the Material and Methods. Cells from MER and LXR were analyzed following one serial passage. Primary untreated tumors generally contained 2 or more non-related stem line populations which frequently differed in the gain and/or loss of whole chromosomes in addition to different clonal markers. In contrast, recurrent tumor samples obtained within a year or less of the primary surgery generally had only a single stem line in the samples we could analyze. The recurrent tumor stem lines also contain very complex karyotypes with multiple abnormalities including non-reciprocal translocations, deletions, duplications and marker chromosomes. Whole chromosomes 7 and/or 22 were over-represented in tumors DI and DIR [10]. In tumors ME and MER we were able to identify at least 2 normal and/or derivative chromosome 7 and 22's in addition to 1–5 marker chromosomes. Chromosome 7 was over-represented in the primary tumor LX while in recurrent tumor LXR only one normal chromosome 7 was present along with 1–3 derivative 7 s. Chromosome 22 was under-represented in the primary and over-represented in the recurrent tumor in addition to fragments within some of the derivative chromosomes and marker chromosomes.

### FISH analyses

**Figure 3:**
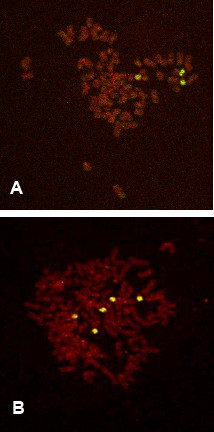
**FISH using whole chromosome paint for chromosome 22**. Whole chromosome paint for chromosome 22 labeled with SpectrumOrange™, showing the presence of chromosome 22 fragments in (A) cell line ME and (B) cell line MER.

To identify the regions of chromosome 22 that were retained in the chromosome 22 fragments in cells from recurrent tumor and cells selected for BCNU resistance *in vitro *we used fluorescent *in situ *hybridization (FISH). We began by confirming previous karyotypic results demonstrating over-representation of part or all of chromosome 22 in cells selected for resistance *in vitro *or *in vivo *(recurrent tumor) using whole chromosome paint (WCP) for chromosome 22. There was an increase in the number of fluorescent signals from chromosome 22 in cells selected for resistance to BCNU *in vitro *or *in vivo *when compared to cells from primary, untreated tumor. Fragments of chromosome 22 were particularly evident in cells from recurrent tumor MER (Figure 3).

**Figure 4:**
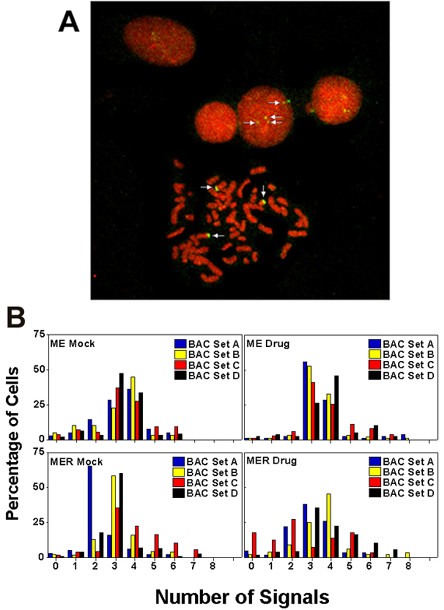
**Over-representation of BAC probes in the ME/MER cell line series**. (A) FISH using BAC pool D as probe on cell line MER. Arrows indicate BAC sequences. (B) Graphs showing over-representation of pooled BAC probes in the ME/MER cell line series.

To identify the region of chromosome 22 that is over-represented in BCNU resistant cells, we began with pooled BAC probes (Figure 1). Whereas over-representation of BAC DNA sequences from all four regions could be found in some cells such as MER, over-representation of sequences from BAC pools C and D was particularly evident in cells from all recurrent tumors, with an increase seen when these cells were further selected *in vitro *for BCNU resistance as shown in Figure 4 for tumors ME/MER. In this figure, the black bars representing BAC pool D can be seen to shift to higher signal numbers when the cells are selected for BCNU resistance. In fact, when cells from recurrent tumor were selected for resistance to BCNU, the region most commonly over-represented corresponded to BACs in the C and D groups (Figure 4). Logistic Regression was used to compare the portion of each of the signals in each group (A, B, C, D) of chromosome 22. Analyses were then done to demonstrate statistical significance of the results of group A & B compared with C & D. PROC logistic regression was used to analyze ME mock versus ME drug and MER mock versus MER drug. The shift of signals from BAC clone groups C & D in ME mock versus MER mock was statistically significant (<.0001). These was also a statistically significant difference between BAC groups A & B versus C & D when ME mock is compared to ME drug (.0122). Finally, the shift in signal number between MER mock and MER Drug was also statistically significant (<.0001).

**Figure 5:**
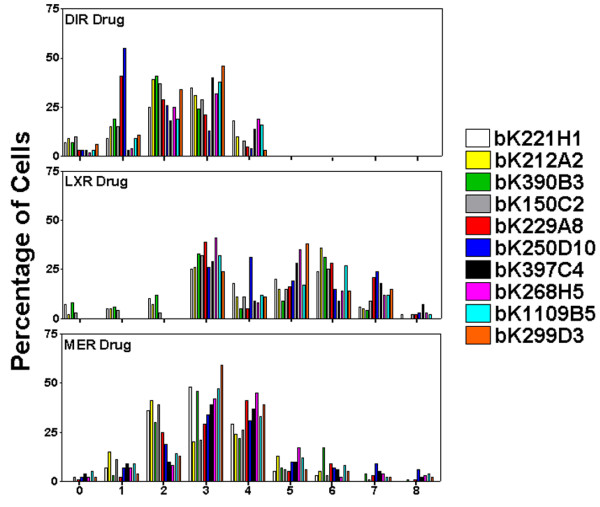
**Copy number of BACs in cells from recurrent tumors resistant to 10 μg/ml BCNU**. Tumor DIR did not have more than 4 signals for any BAC probe.

To further map the region(s) of chromosome 22 retained in cells from recurrent tumor and in cells from recurrent tumor selected for resistance to BCNU, we used individual BACs as FISH probes (Table 3 and Figure 5). This work demonstrated that the region retained most frequently is between BACs bk212A2 and bk299D3, which corresponds to 22q12.3–13.32. BACs outside of this region did not show frequent retention in these cells. Differences in the specific pattern of BAC representation in each cell line (Figure 5) provides evidence that the observed similarities are not due to cross-contamination of the cell lines.

## Discussion

Genetic analyses of primary human malignant gliomas have demonstrated numerous alterations including the over-representation of chromosome 7, and normal or under-representation of chromosome 22 in primary untreated glioblastomas multiforme [18]. Molecular cytogenetic analyses echoed the heterogeneity and complex genetics of these tumors identified by standard cytogenetics. The majority of malignant gliomas recur at or near the site of the original tumor, and it is thought that recurrent tumor arises from cells that survived the patient's treatment. Thus, cells in the recurrent tumor can be thought of as being selected for resistance *in vivo*. This is further suggested by cells in the recurrent tumor that carry markers identified in the primary untreated cells, i.e. del(17)(p11.2) found in the LX and LXR cells shown in Figure 2. We have demonstrated that the heterogeneity present in primary tumors is markedly reduced in recurrent tumors suggesting that among the large number of genetic subpopulations, several may contain resistance and/or survival factors that permit selection and propagation. However, despite the presence of complex karyotypes with multiple abnormalities, cells selected for BCNU resistance *in vitro *or *in vivo *have a specific genetic characteristic – near diploid with over-representation of chromosome 7 and part, or all of chromosome 22 [2,10]. While in recurrent tumors whole copies of chromosome 22 may be under-represented, FISH analysis demonstrated that numerous fragments of chromosome 22 were inserted in derivative and marker chromosomes. This was not found for other chromosomes when analyzed in a similar manner, and additional fragments of all other chromosomes were only randomly retained. To map the region of chromosome 22 that is over-represented in BCNU-resistant cells, we performed FISH analyses using pooled BAC probes. The over-represented region was further defined by use of individual BACs. This work established that this region encompassed 22q12.3–13.32.

When cells from recurrent tumor were further selected *in vitro *for BCNU resistance, the percentage of cells containing the retained region of chromosome 22 increased, suggesting a role in BCNU resistance and/or cell survival for a gene or genes mapped to this region. One gene mapped to this region is the gene encoding the B-chain of platelet-derived growth factor (PDGF-B) [19]. We have previously demonstrated that this gene is over-expressed in cells selected for resistance to BCNU and is probably involved in the autocrine growth of these cells [10]; however, there is no evidence that expression of this gene is directly involved in chemotherapy resistance. Differential mRNA display and cDNA microarray analyses have not demonstrated consistent over-expression of additional specific genes mapped to this area in cells selected for resistance to BCNU. This suggests that sequences in this region may be contributing to a novel mechanism of resistance.

We have recently begun studies of microRNA (miRNA) expression in cells selected for resistance to BCNU. Preliminary results suggest that hsa-let-7b is over-expressed in ME cells selected for BCNU resistance and MER cells before and after selection for BCNU resistance. This miRNA is mapped to chromosome 22q13.31, the region we have found to be over-represented in BCNU resistance cells. Other miRNAs mapped to this region include hsa-let-7a-3 which is just centromeric to let-7b and hsa-mir-33 which is mapped to 22q13.2. Neither of these miRNAs are over-expressed in these cells, suggesting that this is not simply a non-specific results of over-representation. While the precise function of these miRNAs has not been elucidated, the hsa-let7 family has been implicated in the regulation of ras [20], and reduced expression of hsa-let7 is associated with poorer survival in lung cancer [21]. Our current studies are aimed at the identification of the role, if any, of sequences mapped to chromosome 22 in BCNU resistance.

## Conclusion

In summary, we have found that cells selected for resistance to BCNU either *in vitro *or *in vivo *(recurrent tumor) have over-representation of a specific region of chromosome 22 encompassing 22q12.3–13.32. There are no genes known to be involved in resistance mapped to 22q12.3–13.32, suggesting the presence of a gene or DNA sequence involved in a novel mechanism contributing to the growth of resistant cells. Further studies are underway to identify a gene(s) or non-coding RNA on chromosome 22 that is involved in chemotherapy resistance.

## List of abbreviations

BCNU, 1,3-bis(2-chloroethyl)-1-nitrosourea

FISH, fluorescent *in situ *hybridization

BAC, bacterial artificial chromosome

MAB, Waymouth MAB 87/3 medium

FCS, fetal calf serum

WCP, whole chromosome paint

DAPI, 4',6-Diamidino-2-phenylindole

PDGF-B, platelet-derived growth factor, B chain

## Competing interests

The author(s) declare that they have no competing interests.

## Authors' contributions

NCH performed all FISH analyses, statistical analyses and helped draft the manuscript.

JRS supervised the isolation of the cell lines and performed all karyotyping.

ACS conceived the study, directed its design and coordination, obtained and grew the BACs and finalized the manuscript.

All authors read and approved the final manuscript.

## Pre-publication history

The pre-publication history for this paper can be accessed here:



## Figures and Tables

**Table 1:** Patient information

**Tumor code^a^**	**Age^b^**	**Sex**	**Diagnosis^c^**	**Treatment^d^**	**Days between primary and secondary surgery**	**Survival (days)**
DI/DIR	38	F	GBM	Irradiation and BCNU	111	426
LX/LXR	48	M	Gliosarc./GBM^e^	Irradiation and BCNU	203	466
ME/MER	49	F	GBM	Irradiation and BCNU	138	285

^a ^The tumor code is a random 2 letter code. Recurrent tumor from the same patient receives the same code with the addition of R. For each patient the primary and recurrent tumors were diagnosed by the same neuropathologist.

^b ^Age in years at diagnosis of primary tumor.

^c ^GBM = glioblastoma multiforme; gliosarc. = gliosarcoma.

^d ^BCNU = 1,3-bis(2-chloroethyl)-1-nitrosourea.

^e ^Different diagnoses of primary and recurrent tumors. Gliosarcoma is considered a variant of glioblastoma multiforme and the gliomatous and sarcomatous areas have similar genetic profiles and are thought to have a monoclonal origin [22,23]. Tumor LX was primarily glial, with a small sarcomatous element.

**Table 2:** Karyotype analysis of primary/recurrent tumor cells

**Clonal Designation**	**Karyotypic deviation**
DI	45,X,-X,+7,-10[9]/45,idem,-9,der(9)t(9;11)(p22;q21),-11[13]/45,idem,-9,der(9)t(9;11)(p22;q21),-11,-14,-18,del(11) (q21),der(14)t(14;18)(P11;q12),der(22)t(22:?)(p11;?)[44]/90,idem ×2[5]/88,idem ×2,+6,+11,-11,+15,15,+19 [cp12]/85-86,idem,+X,-3,+5,-8,+11,-11,+12,+12,-13,+15,-15,i(15q),-19,+20,-20,+21[31] non-clonal cells [1] 46,XX[13]
DIR	45,X,-X,+7,-9,-10,-11,-14,-18,der(9)t(9;11)(p22;q21),del(11)(q21),der(14)t(14;18)(P11;q12),der(22)t(22:?) (p11;?)[159] non-clonal cells[7]
ME	45,XX,del(9)(p13),add(9)(p21),+9-13,-17[9]/47,idem,+mar1,+mar2,1-3dmin[6]/48,idem,+7[8]/49,idem,+mar3[13]/90,idem×2[5]/80-85,idem×2,-1,+8,-8,-10,+11,-11,+12,-12,-19,-21,-21-22,+mar3,3-7dmin[cp38] non-clonal cells [9] 46,XX[6]
MER	55,X,der(X)t(X:?)(q25;?),der(1)t(1;?)((p32;?),×2,+1,inv(2)(p13q21),der(2)t(2;14)(q23;q13),add(2)(p25.1),+2,add(3) (q29) ×2,+3,-4,del(5)(q13),-5,iso(7)(p11.1),der(9)t(9;1)(p22;p13),del(10)(p11.2), der(10)t(10;?)(q22;?),inv(11)(q13q23)×2,+11,-12,-13,-15, -17,add(18)(q23),add(19)(q13.3), add(20)(q13.3),-22,+mar1,+mar2,+mar3,+mar4,+mar5[16]/56,idem, +7,[36]/57,+7,+mar6 [5] non-clonal cells [23]
LX	56-57,XX,+1,-1,+2,+2,+6,-6,i(6)(p10),+7,+7,+8,+9,der(9)t(9;12)(p13;q11),ins(9;?)(p13;?),+11,+14,+15,+16,-17,del(17)(p11.2),+21,-22,2-3mar[cp11]69,XX,+1,+3, add(3) (q29),x2,+3,+3,+4,del(5)(p13.3p15.1)del(6)q22)×2,+6,+7,+7,+7,del(9)(p13) ×2,+11,+11,+12,+15, +15,-17,-17,+18, +18,+20,+20,+21,+21,+mar3,+mar4,+mar5,[21]/68,idem,-10,-mar3,+mar5[19]/67,idem,-3, -10,+17,-mar3,+mar6[10] non-clonal cells [6]
LXR	46-49,XX, +1, -1, del(1)(p13), del(1)(q12), del(1)(q25), der(?)t(1;?)(q21;?),-2, -2, der(2)t(2;9)(p13;q13),der(2)ins(2;4)(q11.2;12q34), add(2)(q31), -3, add(3)(q21), del(3)(p22;p23),-4, der(4)t(4;8)(q13.2;q11.2), -5, del(5)(p15.1;p15.2), add(5)(q11.1), -6, der(6)t(1;6)(p11;q11), del(6)(q21), add(7)(q21.2), +der(7)t(7;?)(q11.2;?), -9, add(9)(q32), -10, +11, add(11)(p11.2), -12, add(12)(q24.3), -13, add(15)(q21.2), -16, del(17)(p11.2) ×2, add(20)(q11.1), -21, +22, 5, -6, mar[cp41] non-clonal (16)

**Table 3:** Retained BAC DNAs in cells from recurrent tumor selected for resistance to 10 μg/ml BCNU.

Cell Line	# of signals	221H1	212A2	390B3	150C2	229A8	250D10	397C4	268H5	1109B5	299D3
DIR	0	7	9	7	10	3	3	3	2	3	6
	1	9	15	19	15	41	55	3	4	9	11
	2	25	39	41	37	29	26	18	25	19	34
	3	35	31	24	29	21	13	40	32	38	46
	4	18	10		8	5	4	14	19	16	3
LXR	0	7	2	8	3	0	0	0	0	0	0
	1	5	5	6	4	0	0	0	0	0	0
	2	10	7	12	3	0	0	0	0	0	0
	3	25	26	33	32	39	26	29	41	32	24
	4	18	11	5	11	5	31	9	8	12	11
	5	20	15	9	15	16	19	28	35	17	38
	6	24	36	31	25	28	15	9	14	27	14
	7	6	5	4	9	21	24	18	12	12	15
	8	2	0	0	2	2	3	7	3	2	0
MER	0	0	0	0	2	1	2	4	2	5	2
	1	7	15	3	11	2	7	9	7	9	4
	2	36	41	30	39	25	19	10	8	14	13
	3	48	20	46	21	29	34	39	42	47	59
	4	29	24	22	26	41	31	37	45	33	39
	5	5	13	7	6	5	10	10	17	12	6
	6	3	5	17	3	9	7	6	2	8	5
	7	0	0	4	1	3	9	5	4	2	2
	8	0	0	1	0	1	6	2	3	4	2
